# A Novel Role for PX, a Structural Protein of Fowl Adenovirus Serotype 4 (FAdV4), as an Apoptosis-Inducer in Leghorn Male Hepatocellular Cell

**DOI:** 10.3390/v12020228

**Published:** 2020-02-18

**Authors:** Mingliang Zhao, Xueyan Duan, Yongqiang Wang, Li Gao, Hong Cao, Xiaoqi Li, Shijun J. Zheng

**Affiliations:** 1 Key Laboratory of Animal Epidemiology of the Ministry of Agriculture, College of Veterinary Medicine, China Agricultural University, Beijing 100193, China; brightzhao1992@163.com (M.Z.); bestxy@188.com (X.D.); vetwyq@cau.edu.cn (Y.W.); gaoli194@cau.edu.cn (L.G.); caohongxwen@cau.edu.cn (H.C.); 2 College of Veterinary Medicine, China Agricultural University, Beijing 100193, China

**Keywords:** FAdV4, PX, nuclear localization, apoptosis, viral replication

## Abstract

Hydropericardium-Hepatitis Syndrome (HHS) caused by Fowl Adenovirus Serotype 4 (FAdV4) infection is a severe threat to the poultry industry worldwide, especially in China since 2015. Recent studies show that FAdV4 induces liver injury through apoptosis. However, the underlying molecular mechanism is still unclear. We report here that FAdV4 infection caused apoptosis in Leghorn male hepatocellular (LMH) cells and that PX, a structural protein of FAdV4, acted as a major viral factor inducing apoptosis. Furthermore, the nuclear localization of PX is determined by the R/K regions of PX and required for PX-induced apoptosis. Moreover, alanines 11 and 129 of PX are crucial to PX-induced apoptosis. Inhibition of FAdV4-induced apoptosis by caspase inhibitors retarded viral replication, suggesting that PX serves as a virulence factor for FAdV4 infection, which may further our understandings of the pathogenesis of FAdV4 infection.

## 1. Introduction

Adenoviruses are widely-distributed microbes across the globe, and can infect varied species of vertebrates, including birds, animals and humans. Fowl adenoviruses, belonging to the *Aviadenovirus* genus in the *Adenoviridae* family, can be divided into 12 serotypes, including FAdV1-7, FAdV8a/b, and FAdV9-12. Fowl Adenovirus serotype 4 (FAdV4), mainly infects 3- to 6-week old broilers [1]. Hepatitis-hydropericardium syndrome (HHS) was observed in the diseased chickens with FAdV4 infection clinically characterized by swollen livers with focal necroses and petechial hemorrhages, as well as enlarged pericardial sacs containing clear and yellowish fluids. Recently, FAdV4 has caused numerous outbreaks of HHS in chickens in China, leading to severe economic losses to the stakeholders.

FAdV4 is a double-stranded DNA (dsDNA) virus, and its genome, 43.7 kb, encodes approximately 11 structural proteins and 32 non-structural proteins [2]. Currently, there are few reports available regarding the pathogenesis of FAdV4. It was found that Hexon and Fiber 2 genes of FAdV4 HNJZ strain (GenBank ID: KU558760.1) were closely associated with the virulence of the virus [3]. Interestingly, the FAdV4 strains that recently emerged in China had the same deletion of 1966 bp in the genome compared with the non-virulent FAdV4 viruses (ON1 strain) [2]. However, the experimental evidence shows that the natural deficiency of 1966 bp in the viral genome was not essential for the increased virulence of the recently isolated FAdV4 in China [4]. Currently, the virulence-associated factors of FAdV4 responsible for clinical HHS are still unknown.

In comparison with avian adenoviruses, human adenoviruses are very well studied and have been usually used as vectors for gene-therapeutic research [5]. The early genes of human adenoviruses are involved in host-pathogen interactions, including cell cycle progression, host antiviral response, apoptosis and activation of the late promoter for late gene expression [6,7]. Gam-1, an early gene of adenovirus, disrupts PML, whose antiviral function is p53-independent or dependent [8]. However, FAdV1 Gam-1 inhibits TNF-alpha induced apoptosis through the NF-κB signaling pathway in Dark cell lines [9]. It was found that human adenovirus protein X (PX), also named Mu, modulates expression of E2 proteins [10], and is involved in taking the linear double-stranded DNA genome to the capsid during viral replication [11,12], but the role of Fowl adenovirus PX is largely unknown. A recent report indicates that the FAdV4 isolate caused liver injury largely through apoptosis, autophagy and a severe inflammatory response [13]. However, the pathogenesis of FAdV4 infection is still not clear.

In this study, we found that infection of LMH cells by FAdV4 induced apoptosis in LMH cells. By screening for viral components involved in FAdV4-induced apoptosis, we identified PX as a major viral protein inducing apoptosis in host cells. Furthermore, nuclear translocation of PX is required for PX-induced apoptosis, and alanines 11 and 129 of PX are crucial to PX-induced apoptosis. Inhibition of caspase-3 activity by inhibitors suppressed FAdV4 growth in LMH cells.

## 2. Materials and Methods

### 2.1. Cells and Virus

Leghorn male hepatocellular cells (LMH cells), from an immortalized chicken liver cell line, were kindly provided by Dr. Jinhua Liu (CAU, Beijing, China). The LMH cells were cultured in Waymouth’s Medium (MACGENE Technology, Beijing, China) supplemented with 1 × Penicillin-Streptomycin (MACGENE Technology) and 10% fetal bovine serum (Gibco, Grand Island, NE, USA) in a 5% CO_2_ incubator. The cell culture plates were coated with 0.1% gelatin solution (Cat. ES-006-B, Millipore, Billerica, MA, USA) with an addition of 2 mL, and incubated at 4 °C for 5 to 10 minutes before use.

FAdV4 HuBWH strain was isolated from the liver of a diseased chicken with HHS in Wuhan, Hubei province, China in 2016. The isolate was further purified by plaque-forming unit assay (PFU) and stocked at −80 °C.

### 2.2. Reagents, Chemicals, and Antibodies

The jetPRIME^TM^ transfection reagent was obtained from Polyplus-transfection Biotechnology Company (Strasbourg, France) and Genomic DNA Clean kit from ZYMO (Irvine, CA, USA). Annexin V-PE apoptosis detection kit was purchased from BD Pharmingen (Franklin Lakes, NJ, USA), ProLong^TM^ Gold antifade reagent with DAPI from Invitrogen (Carlsbad, CA, USA), and caspase inhibitors z-VAD-fmk, z-DEVD-fmk, z-IETD-fmk, and z-LEHD-fmk from ApexBio (Houston, TX, USA). The pRK5-FLAG plasmid was obtained from Clontech. Anti-GAPDH (60004-1-Ig) antibodies was purchased from Proteintech (Wuhan, China), anti-FLAG M2 (F1804) antibodies from Sigma (City of Saint Louis, USA), anti-Hexon polyclonal antibodies and anti-PX monoclonal antibodies from CAEU Biological Company (Beijing, China), and FITC-conjugated goat anti-mouse IgG, horseradish peroxidase (HRP)-conjugated goat anti-mouse IgG antibodies from DingGuoShengWu (Beijing, China).

### 2.3. Constructions of Recombinant Plasmids

All the genes encoding the indicated FAdV4 proteins were cloned from the FAdV4 HuBWH strain′s genomic DNA. The specific primers used for indicated gene cloning were as follows: For *hexon* (sense primer: 5′ -ATG GCG GCC CTC ACG CCC GAC CTG A- 3′, and antisense primer: 5′ -TTA CAC GGC GTT GCC TGT GGC GAA A- 3′), *fiber1* (sense primer: 5′ -ATG TCG GCC CTA ATC GCC TCC GCA G- 3′, and antisense primer: 5′ -TTA GGG GCC CGG AGC ATT GTT CCC G- 3′), *fiber2* (sense primer: 5′ -ATG CTC CGG GCC CCT AAA AGA AGA C- 3′, and antisense primer: 5′ -TTA CGG GAG GGA GGC CGC TGG ACA G- 3′), *pIIIa* (sense primer: 5′ -ATG AGT TCG ACT GAA GTC TTC GGC G- 3′, and antisense primer: 5′ -TTA GTA AAA GCG TAG GCG CTT GCC T- 3′), *IVa2* (sense primer: 5′ -ATG AGC GCG ATC CCT AAG AAA AGA A- 3′, and antisense primer: 5′ -CTA GGC CTT TTT TGT CTC GTC TGG C- 3′), *pTP* (sense primer: 5′ -ATG GTG CGC ACG CAA CCA GCC CTC G- 3′, and antisense primer: 5′ -CTA GCG GCG CTG TTG GGG ATG GTA T- 3′), *pVI* (sense primer: 5′ -ATG GAC TAC GCC GCC TTG TCG CCT C- 3′, and antisense primer: 5′ -TTA GTA ACA CAA TCG CCT ACT GCT G- 3′), *pVII* (sense primer: 5′ -ATG TCC ATT CTG ATA TCC CCG AAT A- 3′, and antisense primer: 5′ -TCA ACG ACG TCT TCG CAT GCT GCT G- 3′), *pVIII* (sense primer: 5′ -ATG AAC CTC TTG AAC GCC GCA CCC A- 3′, and antisense primer: 5′ -TTA ACC CTG CCA GAA CAC CGG TTC G- 3′), *pX* (sense primer: 5′ -ATG CCC GCC GTG CTT TTG ACC GGG G- 3′, and antisense primer: 5′ -TCA CTT GTT GCC ATA CAA CTT ATT G- 3′) and *penton base* (sense primer: 5′ -ATG TGG GGG TTG CAG CCG CCG ACG T- 3′, and antisense primer: 5′ -CTA CTG CAA GGT CGC GGA ACT CAG A- 3′). Primers were designed with reference to previous publications [2], and synthesized by Sangon Company. All genes and truncated pX (Δ1:1-150aa; Δ2:1-120aa; Δ3:1-90aa; Δ4:91-180aa; Δ5:61-180aa and Δ6:31-180aa) were constructed into the C-terminus of FLAG-tag of pRK5-FLAG by standard molecular biology techniques. Single site mutant pRK5-FLAG-pX (A11T or A129T) and multisite mutant pRK5-FLAG-pX (A11/129T) were constructed with Fast Mutagenesis System obtained from TransGen (Beijing, China).

### 2.4. Apoptosis Assay

LMH cells (1 × 10^6^) were seeded on 12-well plates and cultured for 12 h, followed by infection with FAdV4 or transfection with indicated plasmids (1 μg per well). Twenty-four or forty-eight hours after treatment, cells were harvested, double stained with 7AAD and Annexin V-PE using apoptosis detection kit (BD Pharmingen^TM^), and examined by flow cytometry. The data were then analyzed with CellQuest Pro software, version 5.1 (BD).

### 2.5. Western Blot Analysis

LMH cells (1 × 10^6^) were seeded on 12-well plates and cultured for 12 h, followed by infection with FAdV4 or transfection with indicated plasmids (1 μg per well). Twenty-four or forty-eight hours after treatment, cell lysates were prepared with a lysis buffer (50 mM Tris-HCl, pH 8.0, 150 mM NaCl, 1% TritonX-100, 5 mM EDTA, 10% glycerol, 10 mM dithiothreitol, 1 × completed cocktail protease inhibitor). The sample was boiled with 1 × SDS loading buffer for 10 min. Equal amounts of protein were separated by SDS-PAGE and transferred onto polyvinylidene difluoride (PVDF) membrane. After blocking with 5% skimmed milk, the membranes were incubated with the indicated antibodies. Blots were developed using an ECL kit (Kangwei Biological Company, Suzhou, China).

### 2.6. Confocal Laser Scanning Microscopy Assays

LMH cells (1 × 10^6^) were seeded on 12-well plates and cultured for 12 h, followed by infection with FAdV4 or transfection with indicated plasmids (1 μg per well). Thirty-six hours after treatment, the one-layer cells were fixed with 4% paraformaldehyde, permeabilized with 0.2% Triton X-100 and blocked with 1% bovine serum albumin (BSA). The expressions of FAdV4 PX or FLAG-PX were detected with mouse anti-PX monoclonal antibodies or mouse anti-FLAG monoclonal antibodies, followed by FITC-conjugated goat anti-mouse IgG antibodies. The cells were covered with ProLong^TM^ Gold antifade reagent. The samples were observed under a confocal laser scanning microscope (Olympus Corporation, Tokyo, Japan).

### 2.7. Measurement of FAdV4 Growth in LMH Cells

LMH cells treated with caspase inhibitors or DMSO as a control were infected with FAdV4 at an multiplicity of infection (MOI) of 0.1, and cell cultures were collected at different time points (12, 24, 36, and 48 h) after infection, followed by titration using 50% tissue culture infective doses (TCID_50_) as described previously [14]. Tissue culture wells with a cytopathic effect (CPE) were considered to be positive.

### 2.8. Statistical Analysis

The statistical analysis was performed using GraphPad Prism version 5.0. The significance of the differences between FAdV4 infected cells and controls in apoptosis, between pRK5-FLAG-pX transfected cells and controls in apoptosis, between caspase inhibitor-treated cells and controls in viral growth, were determined by one-way ANOVA.

## 3. Results

### 3.1. FAdV4 Induced Apoptosis in LMH Cells

**Figure 1:**
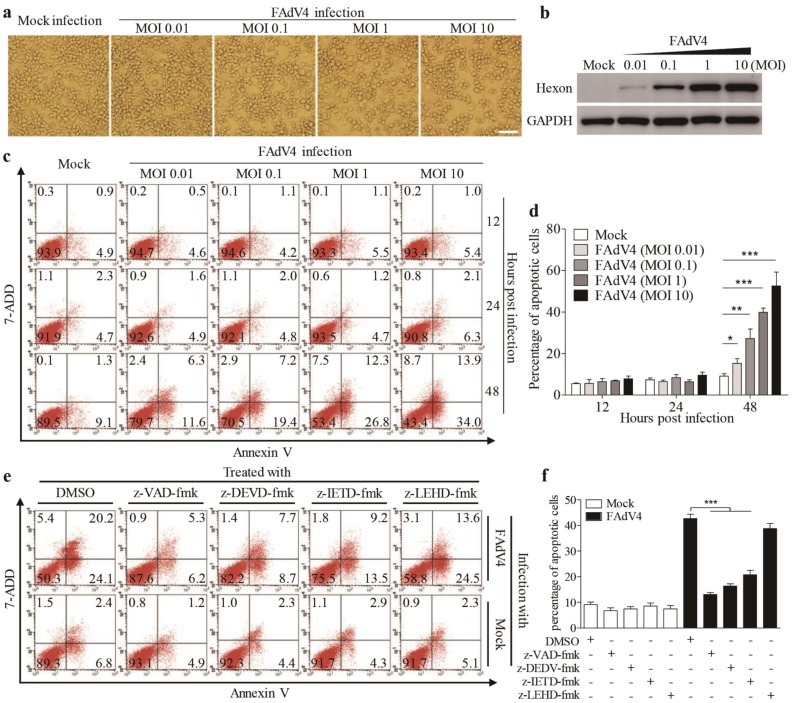
Fowl adenovirus serotype 4 (FAdV4) induced apoptosis in Leghorn male hepatocellular (LMH) cells. (**a**) FAdV4 infection caused a cytopathic effect (CPE) in LMH cells. LMH cells were mock-infected or infected with FAdV4 at an increased MOI of 0.01, 0.1, 1 and 10. Thirty-six hours after infection, LMH cells were visualized under a microscope. The scale bar represents 100μm. (**b**) Examination of the hexon viral protein in FAdV4-infected cells by Western Blot. Forty-eight hours after FAdV4 infection, structural protein hexon was examined with a Western Blot using an anti-hexon specific antibody. (**c**,**d**) FAdV4 infection caused apoptosis in LMH cells. LMH cells were infected with FAdV4 at an increased MOI of 0.01, 0.1, 1 and 10. At different time points (12, 24 and 48h) after infection, cells were harvested, stained with Annexin V-PE and 7-AAD, and analyzed by flow cytometry. Mock-infected cells were used as controls. The percentage of apoptotic cells in (**c**) was quantified (**d**). (**e**) Inhibition of FAdV4-induced apoptosis by caspase inhibitors. LMH cells were seeded on 12-well plates and cultured overnight. Caspase inhibitors (z-VAD-fmk, z-DEVD-fmk, z-IETD-fmk and z-LEHD-fmk) were applied to cell culture for 2h prior to FAdV4 infection. Two hours after infection with FAdV4 at a MOI of 1, cells were cultured with indicated caspase inhibitors again. Forty-eight hours after infection, cells were harvested, stained with Annexin V-PE and 7-AAD, and analyzed by flow cytometry. The percentage of apoptotic cells in (**e**) was quantified (**f**). Results are representative of three independent experiments. Data are presented as mean ± SD, *n* = 3. The significance of differences between the groups was performed by one-way ANOVA (* stands for *p* < 0.05, ** for *p* < 0.01, and *** for *p* < 0.001).

To investigate the pathogenesis of FAdV4 infection, we infected Leghorn Male Hepatocellular (LMH) cells, an immortalized chicken liver cell line, with FAdV4, and examined the cytopathic effects (CPE) in virus-infected cells. As shown in Figure 1a, FAdV4 infection caused severe CPEs in LMH cells in a dose-dependent manner, and the increase of CPE was correlated with the viral growth in host cells, as shown by increased expressions of hexon, a viral structural protein of FAdV (Figure 1b). To further analyze the cell biological features associated with FAdV4-induced CPE in LMH cells, samples stained for Annexin V-PE and 7-AAD were analyzed by flow cytometry. As a result, the number of Annexin V-positive cells markedly increased 48 h after FAdV4 infection compared to that of mock-infected cells (Figure 1c,d), and similar results were obtained for Annexin V and 7-AAD double positive cells. Furthermore, the number of Annexin V-positive cells could be markedly reduced by caspase inhibitors (z-VAD-fmk, pan-caspase inhibitor; z-DEVD-fmk, caspase-3 inhibitor and z-IETD-fmk, caspase-8 inhibitor) except z-LEHD-fmk, the caspase-9 inhibitor (Figure 1e,f). These results demonstrate that infection of LMH cells with FAdV4 induced apoptosis in host cells.

### 3.2. The PX Protein Plays a Major Role in FAdV4-Induced Apoptosis in LMH Cells

**Figure 2:**
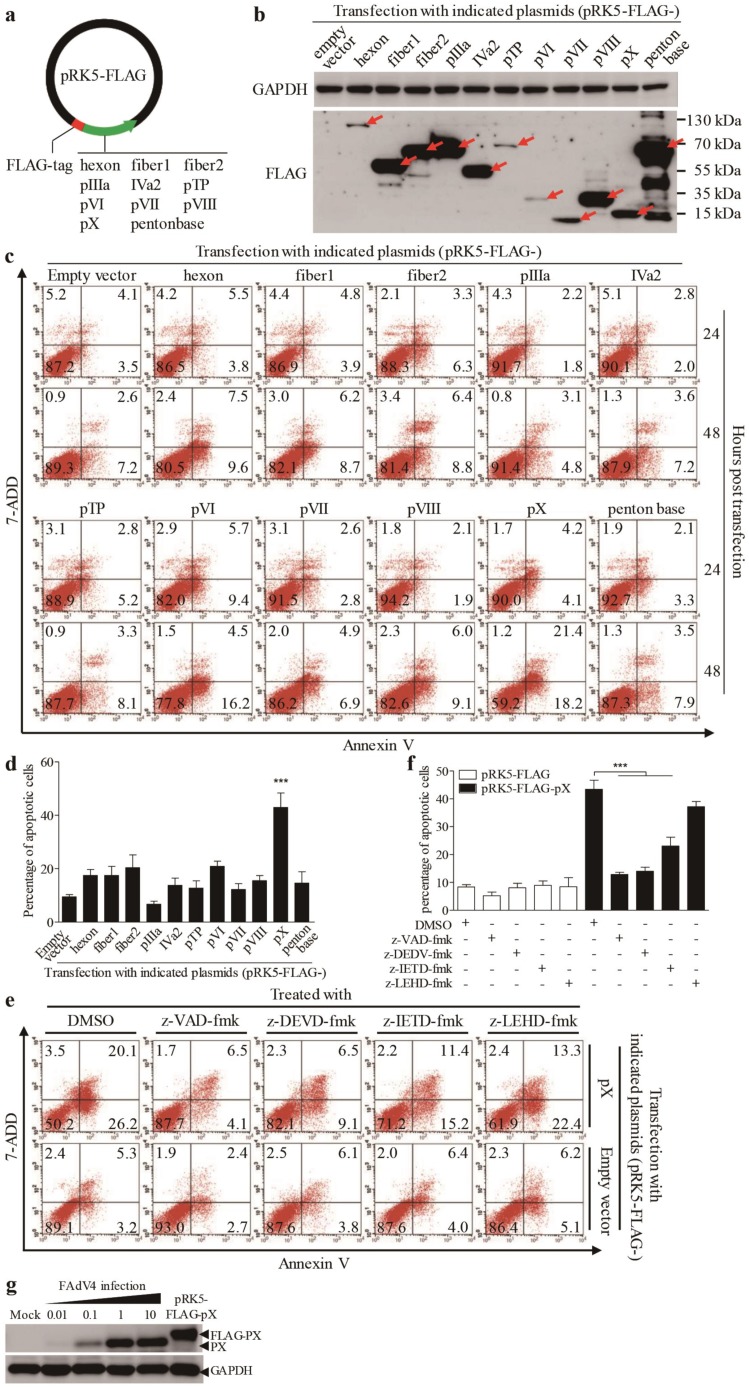
Ectopic expression of PX protein in LMH cells induced apoptosis. (**a**) Schematic diagram of recombinant eukaryotic expression vector pRK5-FLAG-viral genes. We constructed a total of 11 structural proteins into the eukaryotic expression vector pRK5-FLAG. (**b**) Ectopic expressions of FLAG-viral protein recombinants in LMH cells. Recombinant plasmids (pRK5-FLAG-hexon, -fiber1, -fiber2, -pIIIa, -IVa2, -pTP, -pVI, -pVII, -pVIII, -pX and -penton base) were individually transfected into LMH cells using the jetPRIME^TM^ transfection reagent. Forty-eight hours after transfection, cells were harvested and examined with Western Blot using anti-FLAG antibodies. (**c**,**d**) Twenty-four or 48 h after transfection as above described, cells were harvested, stained with Annexin V-PE and 7-AAD, and analyzed by flow cytometry. Empty plasmid-transfected cells were used as controls (**c**). The percentage of apoptotic cells 48 h post-transfection (as in **c**) was quantified (**d**). (**e**) Inhibition of PX-induced apoptosis by caspase inhibitors. LMH cells were seeded on 12-well plates and cultured overnight. Caspase inhibitors (z-VAD-fmk, z-DEVD-fmk, z-IETD-fmk and z-LEHD-fmk) were added to cell culture for 2 h prior to transfection with pRK5-FLAG-pX. Six hours after transfection, cells were again cultured with a caspase inhibitor. Forty-eight hours after transfection, LMH cells were harvested, stained with Annexin V-PE and 7-AAD, and analyzed by flow cytometry. The percentage of apoptotic cells in (**e**) was quantified (**f**). (**g**) Comparison of PX expressions in LMH cells by pRK5-FLAG-pX transfection with that of FAdV4 infection. LMH cells were seeded on 12-well plates and cultured overnight, followed by transfection with pRK5-FLAG-pX at 1μg per well (the same amount as that used in **c**) or infection with FAdV4 at an MOI of 0.01, 0.1, 1 or 10. Forty-eight hours after infection or transfection, cell lysates were prepared and analyzed by Western Blot using an anti-PX specific monoclonal antibody. Results are representative of three independent experiments. Data are presented as mean ± SD, *n* = 3. The significance of the differences between the groups was performed by one-way ANOVA (*** stands for *p* < 0.001).

As FAdV4 infection caused apoptosis in LMH cells, we proposed that one or more components of FAdV4 were responsible for FAdV4-induced cell death. To test this hypothesis, we cloned the genes (*hexon*, *fiber 1*, *fiber 2*, *pIIIa*, *IVa2*, *pTP*, *pVI*, *pVII*, *pVIII*, *pX* and *penton base*) encoding the indicated proteins of FAdV4 and made a FLAG fusion for each of these indicated proteins and expressed them in LMH cells by transfection (Figure 2a,b). Forty-eight hours after transfection, all 11 fusion proteins could be detected in LMH cells. Interestingly, as shown in Figure 2c,d, the number of Annexin V-positive cells significantly increased 48 hours post-transfection with pRK5-FLAG-pX compared to that of controls (*p* < 0.001). Furthermore, the number of Annexin V-positive cells with pRK5-FLAG-pX transfection could be markedly reduced by caspase inhibitors (z-VAD-fmk, z-DEVD-fmk and z-IETD-fmk) but not by z-LETD-fmk (Figure 2e,f). These results indicate that FAdV4 PX is the major viral component responsible for FAdV4-induced apoptosis in LMH cells. To rule out the possibility that the expression of viral protein in cells by transfection may cause artifacts due to the quantity of overexpressed proteins above physiological levels, we compared the quantity of viral PX in FAdV4-infected cells with that of pRK5-FLAG-pX-transfected controls using a Western Blot assay. As a result, the PX expression level in cells transfected with pRK5-FLAG-pX was approximately equivalent to that of viral PX in cells infected with FAdV4 at an MOI of 1 or 10 (Figure 2g). These data suggest that FAdV4 PX serves as a major apoptosis-inducer during FAdV4 infection in LMH cells.

### 3.3. Localization of PX Protein in the Nucleus is Required for PX-Induced Apoptosis

**Figure 3:**
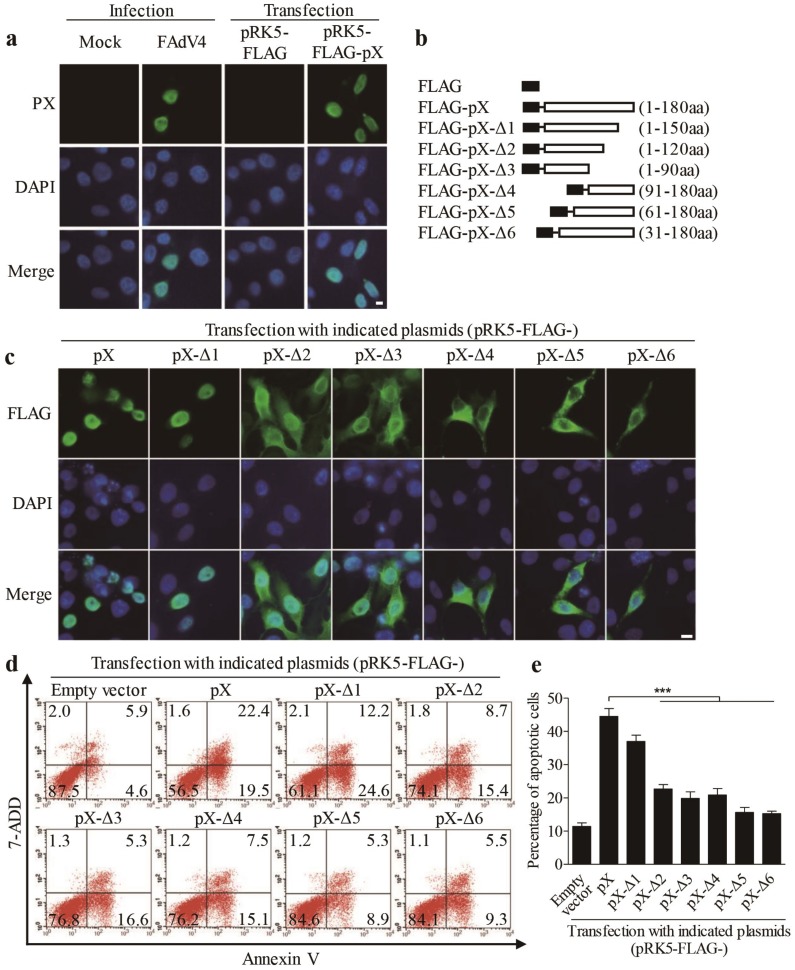
Localization of PX in the nucleus is required for PX-induced apoptosis. (**a**) LMH cells were seeded on 12-well plates and cultured overnight, followed by infection with FAdV4 at a MOI of 1 or transfected with pRK5-FLAG-pX at 1 μg per well. Twenty-four hours after treatment, cells were fixed and probed with an anti-PX monoclonal antibody, followed by incubation with FITC-conjugated goat anti-mouse antibodies (green). Cell nuclei were counterstained with DAPI (blue). Cells were observed with a fluorescence microscope. The scale bar represents 10 μm. (**b**) Schematics represent the genes encoding the full-length or truncated PX molecules (from Δ1 to Δ6). (**c**) Distribution of PX or PX-truncated mutants in LMN cells. LMH cells were transfected with indicated plasmids as described in (**a**). Twenty-four hours after transfection, cells were fixed and probed with mouse anti-FLAG antibodies, followed by incubation with FITC-conjugated goat anti-mouse secondary antibodies (green). Cell nuclei were counterstained with DAPI (blue). LMH cells were observed with a fluorescence microscope. The scale bar represents 10 μm. (**d**,**e**) Examination of apoptosis in LMH cells expressing PX or PX-truncated mutants. LMH cells were transfected with indicated plasmids as described above. Forty-eight hours after transfection, cells were harvested, stained with Annexin V-PE and 7-AAD, and analyzed by flow cytometry (**d**). The percentage of apoptotic cells in (**d**) was quantified and shown in (**e**). Results are representative of three independent experiments. Data are presented as mean ± SD, *n* = 3. The significance of the differences between the groups was performed by one-way ANOVA (*** stands for *p* < 0.001).

As FAdV4 PX plays a major role in FAdV4-induced apoptosis, we attempted to find out the localization of PX in FAdV4-infected cells or cells transfected with pRK5-FLAG-pX using an immunofluorescent antibody assay (IFA). As shown in Figure 3a, PX was localized in the nucleus of cells with both FAdV4 infection and pRK5-FLAG-pX transfection, which prompted us to propose that the nuclear localization of PX might be necessary for PX-induced apoptosis. To test this hypothesis, we constructed a series of PX deletion mutants fused to the FLAG tag (Figure 3b). We expressed these PX deletion mutants (from Δ1 to Δ6) and controls in LMH cells and examined the subcellular localization of these deletion mutants as well as apoptosis in these transfected cells. As a result, PX mutant Δ1(1-150 amino acids), similar to the full-length PX, was merely localized in the nucleus, while mutants Δ2 and Δ3 were distributed in both the nucleus and cytoplasm. In comparison, PX mutants (Δ4-Δ6) were largely accumulated in the cytoplasm (Figure 3c), suggesting that the domains of amino acids 1-30 and 120-150 were associated with the distribution of PX. Interestingly, PX mutant Δ1 retained most of the apoptosis-inducing ability of PX. In comparison, the apoptosis induced by PX mutants Δ2-Δ4 markedly decreased, and little apoptosis was observed in cells with PX mutants Δ5 or Δ6 (Figure 3d,e). These data suggest that apoptosis induced by PX and its mutants is associated with their nuclear localization.

**Figure 4:**
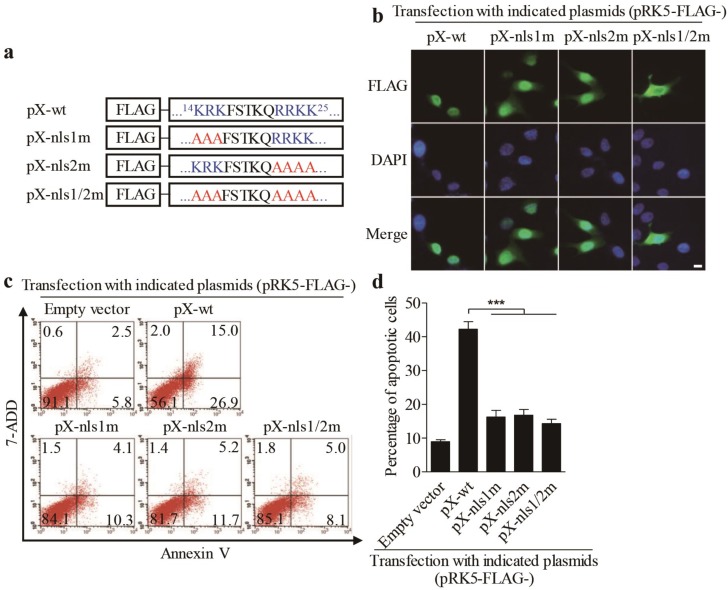
Mutation of nuclear localization sequences (NLS) on both sides of -FSTKQ- within the amino acids 14-25 of PX affected its nuclear translocation associated with inhibition of PX-induced apoptosis. (**a**) Schematics represent the genes encoding the wild-type pX and nuclear localization sequence (NLS) mutant pX molecules (pX-wt, pX-nls1m, pX-nls2m, and pX-nls1/2m). (**b**) Distribution of PX or PX-nls-mutants in LMN cells. LMH cells were transfected with indicated plasmids as described in (**a**). Twenty-four hours after transfection, cells were fixed and probed with mouse anti-FLAG antibodies, followed by incubation with FITC-conjugated goat anti-mouse secondary antibodies (green). Nuclei were counterstained with DAPI (blue). LMH cells were observed with a fluorescence microscope. The scale bar represents 10μm. (**c**,**d**) Examination of apoptosis in LMH cells expressing PX or PX-NLS mutants. LMH cells were transfected with indicated plasmids as described in (**a**). Forty-eight hours after transfection, cells were harvested, stained with Annexin V-PE and 7-AAD, and analyzed by flow cytometry (**c**). The percentage of apoptotic cells in (**c**) was quantified and shown in (**d**). Results are representative of three independent experiments. Data are presented as mean ± SD, *n* = 3. The significance of the differences between the groups was performed by one-way ANOVA (*** stands for *p* < 0.001).

Since PX mutant lacking residues 1–30 amino acids was mainly localized in the cytoplasm, it was intriguing to determine whether the 1–30 amino acids of PX contained nuclear localization sequence (NLS). Therefore, we analyzed the amino acid sequence of PX online (https://www.genscript.com/psort.html). As a result, it was predicted that the domain of 14–25 amino acids was rich with arginines and lysines, suggesting that this domain might be associated with nuclear localization [15]. To confirm the prediction, we replaced critical arginine and lysine residues on both sides of FSTKQ with alanines as indicated in Figure 4a, and the resulting mutants were named PX-nls1m, PX-nls2m, and PX-nls1/2m, respectively. We transfected LMH cells with these pRK5-FLAG-pX mutants and observed the distribution of PX mutant proteins in cells. As shown in Figure 4b, both PX-nls1m and PX-nls2m mutants were more or less localized in the cytoplasm of transfected cells compared to that of PX-wt controls. In contrast, the PX-nls1/2m mutant was largely localized in the cytoplasm, indicating that the domains containing arginine and lysine residues on both sides of FSTKQ were required for PX nuclear translocation. Concurrently, inhibition of PX from nuclear translocation by mutating the nuclear translocation domains could significantly suppress the PX-induced apoptosis (Figure 4c,d), further establishing that localization of PX in the nucleus is required for PX-induced apoptosis.

### 3.4. Amino Acids 11 and 129 Alanines of PX Are Crucial to PX-Induced Apoptosis

**Figure 5:**
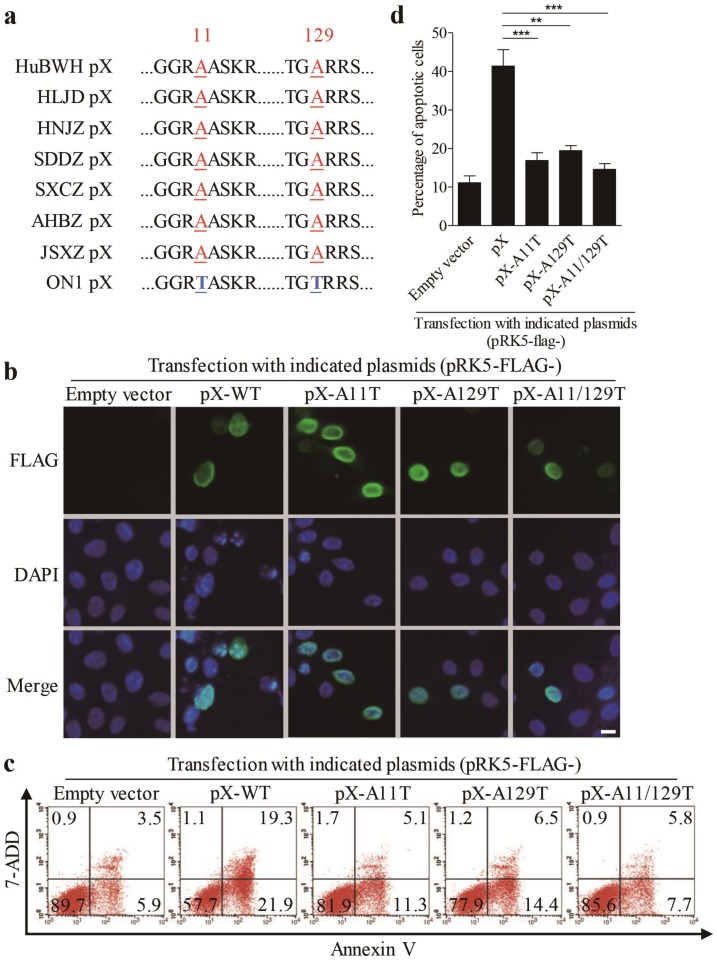
Amino acids 11 and 129 Alanines of PX are crucial to PX-induced apoptosis. (**a**) Alignment of PX amino acid sequences of virulent FAdV4 strains isolated in China with that of apathogenic FAdV ON1 strain. (**b**) Substitution of aa 11 and 129 alanines by threonines did not affect the localization of PX. LMH cells were seeded on 12-well plates and cultured overnight, followed by transfection with vectors pRK5-FLAG-pXA11T, pRK5-FLAG-pXA129T, and pRK5-FLAG-pXA11/129T, respectively. Twenty-four hours after transfection, cells were fixed and probed with mouse anti-FLAG antibodies, followed by incubation with FITC-conjugated goat anti-mouse antibodies (green). Cell nuclei were counterstained with DAPI (blue). Cells were observed with a fluorescence microscope. The scale bar represents 10μm. (**c** and **d**) Examination of apoptosis in LMH cells expressing PX or PX-mutants. LMH cells were transfected with indicated plasmids as described above. Forty-eight hours after transfection, cells were harvested, stained with Annexin V-PE and 7-AAD, and analyzed by flow cytometry (**c**); empty plasmid-transfected cells were used as controls. The percentage of apoptotic cells in (**c**) was quantified and shown as in (**d**). Results are representative of three independent experiments. Data are presented as mean ± SD, n = 3. The significance of the differences between the groups was performed by one-way ANOVA (** stands for *p* < 0.01 and *** for *p* < 0.001).

It was reported that the FAdV4 ON1 strain is an isolate from Canada, and this strain is even in the clustering of ON1 strains that were closer to FAdV4 as determined by phylogenetic analysis, while the chicken infected with ON1 strain showed no clinical signs of inclusion body hepatitis (IBH) or inclusion body hepatitis/hydropericardium syndrome (IBH/HPS) [2]. As PX acts as a major factor responsible for FAdV4-induced apoptosis, we performed the amino acid sequence alignment of ON1 strain PX with that of the virulent FAdV4 strains (including the HuBWH strain, isolated from diseased chickens with IBH by our laboratory) that caused IBH outbreaks in chickens in China. As shown in Figure 5a, there are only two amino acid differences in PX of the ON1 strain (11 and 129 Threonines) compared with that of virulent FAdV4 strains (11 and 129 alanines) in China. Thus, we proposed that the two sites of FAdV4 PX (aa 11 and 129) might be critical to PX-induced apoptosis and that mutations of aa 11 and 129 would, therefore, reduce PX-induced apoptosis. To test this hypothesis, we made mutant constructs pRK5-FLAG-pX-A11T, pRK5-FLAG-pX-A129T, and pRK5-FLAG-pX-A11/129T, transfected these vectors into LMH cells respectively, and examined the localization of these mutants and apoptosis in the transfected cells. As shown in Figure 5b, FLAG-pX-WT and all PX mutants were localized in the nucleus, suggesting that aa 11 and 129 were not relevant to PX localization in cells. However, mutation of A11T, A129T or both A11/129T remarkably reduced apoptosis compared to that of PX-WT control (Figure 5c,d). These results indicate that A11 and 129 are critical amino acids responsible for PX-induced apoptosis, revealing PX as a potential virulence factor for the pathogenic strains of FAdV4.

### 3.5. Inhibition of FAdV4-Induced Apoptosis by Inhibitors Restricted Viral Replication

**Figure 6:**
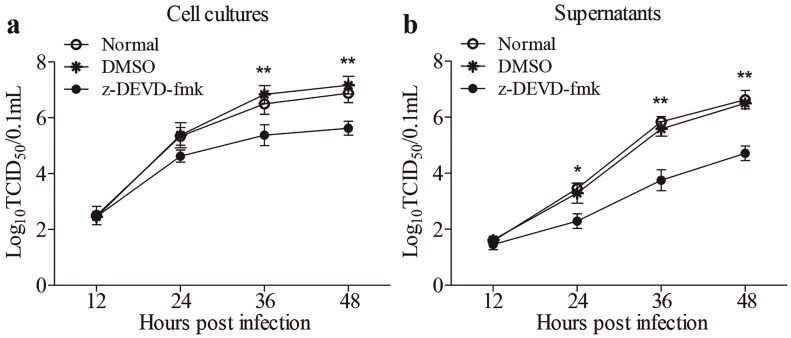
Inhibition of FAdV4-induced apoptosis by caspase-3 inhibitor restricted viral replication. LMH cells treated with z-DEVD-fmk (caspase-3 inhibitor) or DMSO or medium only as controls were infected with FAdV4 at a MOI of 0.1, respectively. At different time points (12, 24, 36, and 48h) post FAdV4 infection, the viral titers in cell cultures (**a**) and supernatants (**b**) were determined with TCID_50_ using 96-well plates. Results are representative of three independent experiments. Data are presented as mean ± SD, *n* = 3. The significance of the differences between the groups was performed by one-way ANOVA (* stands for *p* < 0.05, and ** for *p* < 0.01).

Apoptosis is generally considered as a defense mechanism of host cells in response to pathogenic infection. However, some viruses might take advantage of apoptosis for its spread [16,17]. It would be intriguing to determine whether FAdV4-induced apoptosis is beneficial to viral replication. We treated LMH cells with z-DEVD-fmk, caspase-3 inhibitors inhibiting apoptosis, and examined viral replication at different time points (12, 24, 36 and 48h) post-infection. As a result, the treatment of cells with z-DEVD-fmk markedly reduced viral loads in both cell cultures and the supernatants compared to that of controls (Figure 6a,b), suggesting that apoptosis induced by FAdV4 infection facilitated viral replication.

Taken together, our data show that FAdV4 infection-induced apoptosis in LMH cells, and PX, a structural protein of FAdV4, acts as a major viral factor inducing apoptosis in LMH cells. Furthermore, nuclear translocation of PX is required for PX-induced apoptosis, and aa11 and 129 are crucial to PX-induced apoptosis. Moreover, FAdV4-induced apoptosis facilitates viral replication, suggesting that PX may serve as a virulence factor for FAdV4 infection.

## 4. Discussion

Hydropericardium-Hepatitis Syndrome (HHS) was initially supposed to be caused by nutritional deficiency or toxicity [18]. It was not until 1989 that adenovirus was firstly identified from a purified liver homogenate [19]. The epidemics in the past two decades have provided some evidence confirming that the fowl adenovirus causing HHS worldwide were identified as serotypes 4 and 10 [20,21]. In addition to 3-5-week-old broilers, other avian species, including hens, ducks, pigeons, and quails, could also be infected by FAdV on some occasions [22]. The mortality of this disease could reach up to 80% of susceptible chickens in China [23]. Thus, HHS remains a severe threat to the stakeholders.

It was reported that FAdV4 induced liver injury through apoptosis, autophagy, and an acute inflammatory response [13]. However, the molecular mechanism underlying FAdV4-induced apoptosis is unclear. The genome of FAdV4 potentially contains 46 ORFs encoding proteins as determined by the BD-based Gene Identification tool [2]. In this study, we cloned all 11 proteins existing in the mature capsid with reference to the previous publications [24,25]. Leghorn Male Hepatocellular (LMH) cells are an immortalized chicken hepatic cell line that is typically used for FAdV4 infection and replication, and importantly notable cytopathic effect (CPE) was observed in FAdV4-infected LMH cells. In this study, our data first show that FAdV4 infection caused marked apoptosis in LMH cells. Second, the expressions of viral proteins in LMH cells indicate that PX was a major factor causing apoptosis in cells. Of note, the expression levels of different recombinant proteins in transfected cells may vary due to some unknown reasons although the doses of plasmids are equally transfected in the assay. In this study, we observed that the expressions of Hexon, pTP and pVI were lower than that of the others, which might affect their apoptotic activity. However, we found that the transfection of cells with increasing doses of these plasmids did not change the conclusion. We used this method to screen for apoptosis-inducing viral proteins as candidates in the beginning, but these candidate proteins must be further examined for their apoptotic activities using different methods. We found that the PX expression level in cells transfected with pRK5-FLAG-pX was approximately equivalent to that of viral PX in cells infected with FAdV4 at a MOI of 1 or 10 (Figure 2g). Importantly, the apoptosis-inducing effect of PX strictly depends on its localization in the nucleus as well as A11 and A129 of PX. Furthermore, FAdV4-induced apoptosis facilitates viral replication. These results suggest that PX may serve as a virulence factor for pathogenic FAdV4 strains.

In human adenovirus (HAd), PX, also known as Mu, modulates the expression of E2 protein [12], and plays an important role in attaching the linear double-stranded DNA genome to the capsid during replication [10]. However, the low homology (34.2%) of the PX amino acid sequence between HAd2/5 (GenBank ID: AC_000007.1/AC_000008.1) and FAdV4 (GenBank ID: NC_015323.1) indicates that the function of FAdV4 PX may vary from that of HAd. Our data indicate, for the first time, FAdV4 PX as a major viral protein inducing apoptosis in host cells, which may serve as a virulence factor for pathogenic FAdV4. In addition to PX, other structural proteins of FAdV4, such as Hexon and Fiber 2, were found to be the crucial factors determining the pathogenicity of FAdV4 [3,26,27]. It was reported that Hexon or Fiber2 of FAdV4 HNJZ strain caused apoptosis to more or less degree. Our data show that FAdV4 PX induced much greater apoptosis in LMH cells than Hexon or Fiber2.

Adenovirus assembly takes place in the nucleus of the infected cell [28]. The capsid proteins surrounding the genome DNA must be transported into the nucleus of a host cell to complete the assembly. The 100K-chaperone protein of adenovirus assists the nuclear localization and folding of the hexon protein and acts as a scaffold to facilitate assembly of the hexon trimer [29]. The penton capsomers consisting of a pentameric penton base and trimeric fiber assemble in the cytoplasm and translocate to the nucleus for virion assembly [30]. Both nuclear localization sequences (NLS), the primary amino acid motifs, even secondary or tertiary structural elements in many proteins or RNAs, and the chaperones were fully recognized as the nucleocytoplasmic transport mechanism using specific cellular factors or macromolecular complexes to regulate trafficking of cargoes [31,32]. CCT7, a cytosolic chaperone protein, was determined to interact with Hexon of FAdV4 and was required for viral replication [26], but the effects of CCT7 on Hexon localization remain unknown. Interestingly, we found that both viral PX of FAdV4 and FLAG-PX recombinants expressed in LMH cells were localized in the nucleus and the R/K regions in amino acid residues of 14 to 16 and of 22 to 25 of PX were crucial to the nuclear translocation of these PXs. Furthermore, our data indicate that the nuclear translocation of PX is required for PX-induced apoptosis in cells. It seems that localization of PX in the nucleus is a must for PX-induced apoptosis in host cells.

In recent years, several virulent FAdV4 strains were isolated and identified, including HNJZ, SDDZ (GenBank ID: KU558761.1), SXCZ (GenBank ID: KU558762.1), AHBZ (GenBank ID: KU569295.1), JSXZ (GenBank ID: KU569296.1) and HuBWH in China [33]. These virulent FAdV4 strains had an identical amino acid sequence of PX. Interestingly, we found that all these Chinese FAdV4 stains had the same spontaneous amino acid substitutions at residues 11 (T to A) and 129 (T to A) in the PX protein when compared with non-pathogenic FAdV4 ON1 strain. Surprisingly, mutation of A11T, A129T or both A11/129T back, referring the PX sequence of non-pathogenic FAdV4 ON1 dramatically reduced apoptosis compared to that of virulent FAdV4 PX, indicating that aa 11 and 129 alanines are critical for PX-induced apoptosis. It seems that PX may serve as a virulence factor for pathogenic FAdV4 isolates because inhibition of apoptosis by caspase inhibitors significantly reduced the viral growth in LMH cells. Thus PX-induced apoptosis contributed at least in part to FAdV4-induced apoptosis, which facilitates viral replication. Up to now, some researchers have established a reverse genetic system to generate recombinant FAdV4 [34] or used the CRISPR/cas9 system to delete or insert some nucleotide fragments directly [4]. Therefore, more efforts will be required to investigate the role of PX in the pathogenesis of FAdV4 infection.

In summary, our data show that FAdV4 infection-induced apoptosis in LMH cells and PX acts as a major viral factor inducing apoptosis in host cells. Furthermore, nuclear translocation of PX is required for PX-induced apoptosis, and alanines 11 and 129 of PX are crucial to PX-induced apoptosis. Moreover, FAdV4-induced apoptosis facilitates viral replication, suggesting that PX serves as a virulence factor for FAdV4 infection, which may provide a new clue to the design of a novel vaccine for the control of FAdV4 infection.
